# Vascular and Inflammatory High Fat Meal Responses in Young Healthy Men; A Discriminative Role of IL-8 Observed in a Randomized Trial

**DOI:** 10.1371/journal.pone.0053474

**Published:** 2013-02-06

**Authors:** Diederik Esser, Els Oosterink, Jos op 't Roodt, Ronald M. A. Henry, Coen D. A. Stehouwer, Michael Müller, Lydia A. Afman

**Affiliations:** 1 Top Institute Food and Nutrition, Wageningen, The Netherlands; 2 Nutrition, Metabolism, and Genomics Group, Division of Human Nutrition, Wageningen University, Wageningen, The Netherlands; 3 Department of Internal Medicine and Cardiovascular Research Institute Maastricht, Maastricht University, Maastricht, The Netherlands; Paris Institute of Technology for Life, Food and Environmental Sciences, France

## Abstract

**Background:**

High fat meal challenges are known to induce postprandial low-grade inflammation and endothelial dysfunction. This assumption is largely based on studies performed in older populations or in populations with a progressed disease state and an appropriate control meal is often lacking. Young healthy individuals might be more resilient to such challenges. We therefore aimed to characterize the vascular and inflammatory response after a high fat meal in young healthy individuals.

**Methods:**

In a double-blind randomized cross-over intervention study, we used a comprehensive phenotyping approach to determine the vascular and inflammatory response after consumption of a high fat shake and after an average breakfast shake in 20 young healthy subjects. Both interventions were performed three times.

**Results:**

Many features of the vascular postprandial response, such as FMD, arterial stiffness and micro-vascular skin blood flow were not different between shakes. High fat/high energy shake consumption was associated with a more pronounced increase in blood pressure, heart rate, plasma concentrations of IL-8 and PBMCs gene expression of IL-8 and CD54 (ICAM-1), whereas plasma concentrations of sVCAM1 were decreased compared to an average breakfast.

**Conclusion:**

Whereas no difference in postprandial response were observed on classical markers of endothelial function, we did observe differences between consumption of a HF/HE and an average breakfast meal on blood pressure and IL-8 in young healthy volunteers. IL-8 might play an important role in dealing with high fat challenges and might be an early marker for endothelial stress, a stage preceding endothelial dysfunction.

**Trial Registration:**

ClinicalTrials.gov NCT00766623

## Introduction

A lifestyle factor known to be important in development and progression of cardiovascular disease (CVD) is diet. Although several dietary components and patterns have been related to vascular function, the postprandial response has gained specific attention since it has been associated with an impaired vascular function, low grade inflammation and increased cardiovascular risk [1], [2], [3]. Postprandial effects on vascular function and inflammation are reversible and temporally, but can be of importance since most individuals are in the postprandial state the greater part of the day [4], [5], [6], [7], [8]. Most studies that investigated the postprandial vascular response used flow mediated dilatation (FMD) as measure of vascular function. Vascular function can also be assessed by other measures, of which some have been applied in postprandial studies [9], [10], [11], [12]. Postprandial challenges often used in relation to CVD are high fat (HF) meals, as these atherogenic meals provide a direct source of stress [13]. However, many previous studies only investigated a small part of the postprandial response or were performed in older individuals or in populations with a progressed disease state, such as diabetes, metabolic syndrome, hypertension or cardiovascular disease. The postprandial impact of high fat/high energy (HF/HE) meals on both vascular function and inflammation in healthy young subjects has been less well studied. In addition, many postprandial studies compared HF/HE meals with water consumption, with other macronutrients or only with baseline recordings. In these studies the question remains whether observed postprandial changes are due to the high fat content itself, to a common meal effect, or to circadian rhythm influences. Hence, it is not completely clear whether the HF/HE meal-induced changes in vascular function and inflammation are solely caused by the HF content or by the meal itself and whether this temporary impairment is also elicited in younger healthy individuals. To address these points, we examined the postprandial response after a HF/HE shake in young healthy men on several measures of vascular function and blood markers of endothelial function and inflammation, and compared this response with an average breakfast milkshake. Since most functional measures of vascular function are known to have large variations in reproducibility, the effects of both shakes were studied three times within the same individual.

## Methods

The protocol for this trial and CONSORT checklist are available as supporting information (Protocol S1 and Checklist S1).

### Ethics Statement

All subjects gave written informed consent and the study was approved by the Medical Ethics Committee of Wageningen. The study was conducted according to the principles of the Declaration of Helsinki and in accordance with the Medical Research Involving Human Subjects Act (WMO) and registered at ClinicalTrials.gov (Identifier: NCT00766623).

### Subjects

Twenty healthy male volunteers of Western European descent, between 18 and 27 years, were recruited. Exclusion criteria were a body mass index (BMI) <18 or >28 kg/m^2^, urine glucose concentrations >0,25 g/l, fasting blood glucose <3 or >5.5 mmol/L or blood Hb values <8.4 mmol/L and smoking. Furthermore, subjects were excluded if they were diagnosed with any long-term medical condition or high blood pressure (systolic BP> 140 mmHg and/or diastolic BP>90 mmHg).

### Study design

The study was a double-blind randomized cross-over intervention study in which participants visited the university six times in total; three times to obtain postprandial responses on a HF/HE shake and three times to obtain postprandial responses on an average breakfast shake. The latter was used to acquire a common postprandial response and shakes were therefore not isocaloric. A one-week washout period was the minimum between consecutive study days. Shakes were assigned alternately over the study days and order of start was randomly assigned. A research assistant generated the random allocation sequence. Shakes were given a code and both subjects and researchers were blinded to the intervention. Prior to each study day, subjects consumed a standardized low fat evening meal, were refrained from alcohol or strenuous exercise and were not allowed to eat or drink anything except water after 08.00 pm. For each subject, starting time of every study day was kept constant.

A study day was executed as follows: upon arrival, a cannula was placed, baseline fasted blood samples were collected baseline vascular measurements were done. Subsequently, the subject received either a HF/HE or an average breakfast shake. Postprandial vascular measurements and blood samples were taken 3 and 6 hours after milkshake consumption. These time points were chosen because in previous studies, a maximal FMD response was observed 3 hours after high fat meal consumption and the FMD measure was back to baseline values 6 hours postprandially [14], [15]. Throughout the study day, subjects were not allowed to eat or drink anything except water.

### Shakes

The HF/HE shake consisted of 53%(w/v) fresh cream, 3%(w/v) sugar and 44%(w/v) water and reflected a macronutrient composition of 6g protein, 95g fat (of which 54g saturated), 22g carbohydrates and represented a total energy content of 3992KJ. The average breakfast shake was, based on macronutrient composition, comparable to a breakfast as averagely consumed by young men in the Netherlands [16]. This average breakfast shake consisted of 43%(w/v) full cream milk, 48%(w/v) full cream yoghurt, 4%(w/v) lemonade, 4%(w/v) fantomalt (Nutricia B.V., the Netherlands) and 1%(w/v) wheat fiber and reflected a macronutrient composition of 17g protein, 14.5g fat, (of which 9g saturated), 49.5g carbohydrates and 2.3g fiber and represented a total energy content of 1674KJ (NEVO 2006). Both shakes had a total volume of 500 ml.

### Metabolic parameters

Plasma triacylglycerol (TAG), free fatty acids (FFA), insulin and glucose concentrations were assessed at baseline and 1, 2, 3, 4, and 6 hours after milkshake consumption and were measured by a hospital laboratory (SHO, Velp, the Netherlands).

### Functional measures of vascular function

Measurements of vascular function included micro-vascular skin blood flow, arterial stiffness and FMD. Measures were performed in above mentioned order and whole data was acquired at baseline and 3 and 6 hours after shake consumption. All measurements were performed in supine position after 10 minutes rest, in a quiet temperature controlled room at moderate light intensity.

#### Blood pressure

Blood pressure (BP) ad heart rate (HR) were assessed automatically (DINAMAP® PRO 100) during the functional measurements with a 5 minute interval.

#### Iontophoresis laser Doppler

Micro-vascular skin blood flow was assessed by laser Doppler iontophoresis [17]. Briefly, two ion chambers (MIC-ION6, Moor Instruments, UK) on the volar aspect of the forearm were filled with 1% sodium nitroprusside (SNP) (Sigma – 31444-50G) or 1% acetylcholine (Ach) (Sigma – A6625-25G) solution and iontophoretically administered (MIC2™, Moor Instruments, UK). Skin blood flow was recorded by laser Doppler (MoorFLPI, Moor Instruments, UK) and expressed as incremental area under the curve (AUC) calculated from mean flux outcomes plotted against total recorded time period (NCSS software v07.1.4).

#### Pulse wave analysis of the radial artery

Arterial stiffness was assessed by pulse wave analysis (PWA) of the radial artery by applanation tonometry (SphygmoCor®^CP^ System, ATcor Medical). In short, a pressure-sensitive probe was placed on the radial artery to generate a pulse pressure wave (Sphygmocor software v8.0). In combination with the brachial blood pressure measurements we deduced central aortic pressures and the heart rate corrected augmentation index (AIX) [18].

#### Flow-mediated dilatation

FMD was performed according to techniques described by the International Brachial Artery Reactivity Task Force [19]. In short, after baseline recordings, a pressure cuff on the forearm was inflated and kept constant at a pressure of 200 mmHg for 5 min. Thereafter, the cuff was released and records of the artery were made every 20 seconds for 4 minutes (Picus, ART.LAB v2.1, Esaote benelux bv.). FMD was computed as maximum vessel diameter after cuff release divided by baseline and expressed in percentage. A nitroglycerin dose was administrated sublingually by spray at the end of each day.

### Blood measures

#### Plasma markers

Baseline and postprandial plasma cytokine concentrations were determined once for each shake and analyzed on preformatted arrays on a SECTOR Imager 2400 reader (Meso Scale Diagnostics, LLC) as described previously [20].

#### PBMC Gene expression

Peripheral blood mononuclear cells (PBMCs) were isolated by BD Vacutainer Cell Preparation Tubes. RNA was isolated (RNeasy Micro kit, Qiagen, Venlo, the Netherlands), quantified (Nanodrop ND 1000, Nanodrop technologies, Wilmington, Delaware USA) and quality was determined (Agilent 2100 Bioanalyser, Agilent Technologies, South Queensferry, UK). RNA with a RIN score >7 was thereafter reverse transcribed (cDNA synthesis kit, Promega, Leiden, the Netherlands) and analyzed by qPCR (SensiMix SYBR No-ROX, Bioline, London, UK) on a CFX384 Real-Time System (C1000 Thermal Cycler, Biorad, Veenendaal, The Netherlands). Primer sequences were chosen based on the sequences available in Primer3 (v. 0.4.0). Data was normalized by the housekeeping gene hUPO.

#### Statistics

Study outcomes of all three testing days are expressed as pooled mean and SD was calculated by the root mean squared error. Statistical comparisons were performed by linear mixed models for repeated measures (PASW statistics 17.0.3), using ‘diet’, ‘time point’ and ‘diet x time point’ as fixed effects and subject as random effect. Postprandial responses on plasma cytokines were determined once for each shake and baseline values were included as covariate in the model if they were of significant influence and Studentized residuals >3, obtained from the mixed model, were considered outliers and removed from the model. A value of P<0.05 was considered significant.

## Results

### Subjects characteristics

**Figure 1:**
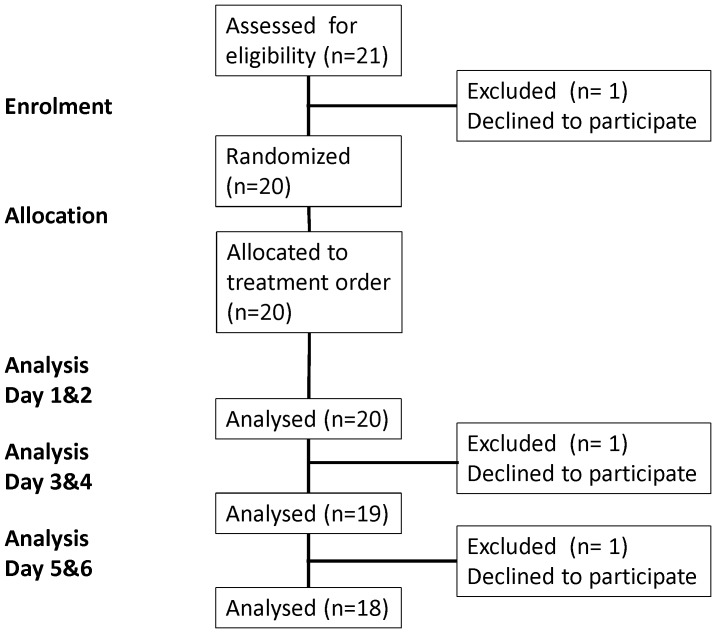
CONSORT flow diagram.

Twenty volunteers entered the study. Eighteen completed all six study days, one volunteer completed four study days and one completed two study days **(** **Figure 1** **)**. Baseline characteristics of the subjects are listed in **Table 1**.

**Table 1:** Subject characteristics.

	Mean ± SD (n = 20)
Age (yrs)	22±2
Length (cm)	185±7
Weight (kg)	78±10
BMI (kg/m^2^)	22.7±2.4
Glucose (mmol/L)	4.9±0.3
TAG (mmol/L)	1.1±0.3
FFA (mmol/L)	0.52±0.18
Insulin (uIU/ml)	6.94±3.22
Systolic BP (mmHg)	111±9
Diastolic BP (mmHg)	59±6

Values are expressed as mean ± SD. Triglycerides (TAG), free fatty acids (FFA), blood pressure (BP).

### Metabolic parameters

Baseline and postprandial changes in TAG, FFA, insulin and glucose are listed in **Table 2** **.** A significant difference in response between the HF/HE and the average breakfast shake was observed for all parameters.

**Table 2:** Baseline and postprandial concentrations of metabolic plasma parameters after high fat/high energy (HF/HE) or average breakfast control shake consumption.

	Shake	Time						P-value		
		Baseline	1h	2h	3h	4h	6h	Shake	Time	Interaction Shake*Time
TAG	HF/HE	1.0±0.2	1.1±0.2	1.5±0.3	1.7±0.3	2.0±0.4	1.5±0.3	<0.001	<0.001	<0.001
(mmol/L)	Control	1.0±0.2	1.0±0.2	1.1±0.2	1.0±0.2	1.1±0.2	0.9±0.2			
Glucose	HF/HE	4.2±0.4	4.0±0.4	3.8±0.3	4.0±0.2	4.1±0.3	3.9±0.3	0.892	<0.001	0.019
(mmol/L)	Control	4.2±0.3	4.1±0.6	3.6±0.4	4.0±0.2	4.0±0.3	4.0±0.3			
FFA	HF/HE	0.50±0.15	0.32±0.10	0.34±0.06	0.55±0.09	0.67±0.07	0.76±0.10	<0.001	<0.001	<0.001
(mmol/L)	Control	0.51±0.17	0.15±0.05	0.17±0.04	0.32±0.09	0.53±0.10	0.78±0.14			
Insulin	HF/HE	6.9±1.8	20.0±11.5	10.2±2.9	7.1±2.5	8.2±2.9	4.8±1.4	<0.001	<0.001	<0.001
(uIU/ml)	Control	7.0±2.3	43.3±17.0	10.9±7.6	5.6±1.8	4.4±1.4	3.3±1.1			

Values are pooled mean ± SD of all three study days (n = 20). Triacylglycerol (TAG), free fatty acids (FFA).

### Measures of vascular function

Outcomes of all functional vascular measures are listed in **Table 3**. Consumption of both shakes resulted in a small but significant postprandial reduction in FMD% at 3 hours that returned to baseline after 6 hours. No significant difference in response between shakes was observed. Consumption of both shakes resulted in an significant postprandial increase in HR and systolic blood pressure (SBP), with a significant higher response after HF/HE shake consumption. No significant postprandial changes were observed for diastolic blood pressure (DBP). Similar to the effects on SBP, HF/HE consumption also resulted in a higher postprandial increase in central aortic pulse pressure. AIX was significantly decreased for both shakes, with no difference in response between shakes. Micro-vascular blood flow was significant reduced after consumption of both shakes, with no difference in response between shakes. Similar results were observed for both SNP and Ach treatment.

**Table 3:** Baseline and postprandial values of vascular function measures after high fat/high energy (HF/HE) or average breakfast control shake consumption.

	Shake	Time				P-value		
		Baseline	3h	6h		Shake	Time	Interaction Shake*Time
**Iontophoresis**								
Total Ach (AUC)	HF/HE	2674±982	1328±576	1175±533	0.597		<0.001	0.451
	control	2464±1030	1457±861	1134±664				
Total SNP (AUC)	HF/HE	2964±823	2189±809	2149±787	0.217		<0.001	0.260
	control	2731±960	2328±868	1997±1091				
**Brachial blood pressure**								
Systolic BP	HF/HE	110±5	114±4	116±4	0.001		<0.001	0.003
(mmHg)	control	110±4	111±4	113±4				
Diastolic BP	HF/HE	59±5	58±4	58±4	0.565		0.124	0.832
(mmHg)	control	59±4	58±4	58±4				
Heart rate	HF/HE	54±5	58±4	57±4	0.001		<0.001	0.001
(BPM)	control	55±4	55±4	55±4				
**Pulse Wave Analysis**								
Central systolic BP	HF/HE	92±4	93±4	94±3	0.001		0.070	0.075
(mmHg)	control	92±4	91±3	92±3				
Central Pulse	HF/HE	33±3	35±2	36±2	<0.001		<0.001	0.028
pressure (mmHg)	control	33±3	33±2	34±3				
AIX (%)	HF/HE	−13±10	−15±10	−16±6	0.012		0.106	0.903
	control	−16±7	−17±7	−18±8				
**FMD**								
Baseline vessel	HF/HE	4212±145	4233±135	4223±135	0.060		0.671	0.519
diameter (µm)	control	4207±140	4180±125	4194±177				
Maximum vessel	HF/HE	4419±179	4422±150	4445±187	0.025		0.026	0.404
diameter (µm)	control	4407±170	4347±134	4410±160				
FMD (%)	HF/HE	5.05±2.01	4.54±1.88	5.36±2.55	0.322		0.004	0.818
	control	4.87±2.50	4.13±2.02	5.25±2.09				

Values are pooled mean ± SD of all three study days (n = 20). Acetylcholine (Ach), sodium nitroprusside (SNP), area under the curve (AUC), beats per minute (BPM), blood pressure (BP), augmentation index (AIX), flow mediated dilatation (FMD).

### Plasma cytokines

Plasma cytokines levels before and after milkshake consumption are listed in **Table 4** **.** A significant increase in IL-8 was observed after HE/HF shake consumption compared to an average breakfast. Plasma concentrations of SAA and VCAM-1 were significantly decreased after a HE/HF milkshake compared to an average breakfast. Consumption of both shakes resulted in a significant postprandial increase in plasma levels of IL-6, whereas E-selectin and thrombomodulin were decreased postprandially.

**Table 4:** Baseline and postprandial changes in inflammatory cytokines after high fat/high energy (HF/HE) or average breakfast control shake consumption.

	Shake	Time			P-value		
		Baseline	3h	6h	Shake	Time	Interaction Shake*Time
CRP	HF/HE	464±615	435±573	412±528	0.150	0.305	0.186
(ng/ml)	control	461±746	641±1357	769 ±1841			
SAA	HF/HE	497±277	422±267	382±206	0.065	0.225	0.022
(ng/ml)	control	520±432	532±490	585 ±604			
sICAM-1	HF/HE	181±27	179±26	176±30	0.464	0.056	0.856
(ng/ml)	control	177±27	179±31	178±32			
sVCAM-1	HF/HE	305±37	296±35	296±35	0.003	0.175	0.038
(ng/ml)	control	294±45	298±49	304±50			
E-selectin	HF/HE	6.03±1.91	5.66±1.81	5.79±1.87	0.744	0.038	0.832
(ng/ml)	control	6.40±2.52	5.83±1.83	5.81±1.60			
P-selectin	HF/HE	51.9±16.1	52.0±15.5	53.0±18.8	0.786	0.912	0.623
(ng/ml)	control	50.5±11.9	51.4±11.0	49.8±13.2			
sICAM-3	HF/HE	1.47±0.28	1.48±0.26	1.52±0.27	0.064	0.042	0.105
(ng/ml)	control	1.52±0.33	1.42±0.18	1.50±0.28			
Thrombomo	HF/HE	2.34±0.42	2.23±0.36	2.21±0.37	0.739	<0.001	0.955
dulin ng/ml)	control	2.39±0.40	2.28±0.39	2.24±0.37			
IL-1β	HF/HE	0.47±0.31	0.47±0.28	0.49±0.32	0.346	0.149	0.712
(pg/ml)	control	0.38±0.17	0.54±0.48	0.57±0.50			
IL-6	HF/HE	0.74±0.24	1.18±0.48	1.38±0.73	0.401	<0.001	0.141
(pg/ml)	control	1.10±1.01	1.40±0.85	2.31±2.31			
IL-8	HF/HE	4.08±0.95	4.36±1.18	4.45±1.00	0.002	0.481	0.043
(pg/ml)	control	4.15±1.20	3.84±0.74	3.78±0.77			
TNFα	HF/HE	6.12±1.70	6.07±1.96	6.12±1.91	0.296	0.677	0.482
(pg/ml)	control	5.90±1.57	6.19±1.75	6.19±1.87			

Values are mean ± SD from the last study days (n = 20).

### PBMC gene expression

We examined expression changes of a selection of genes **(** **Table 5** **)**, known to be involved in inflammation or endothelial function in PBMCs. HF/HE shake consumption resulted in a higher postprandial up regulation of IL-8 after 3 and 6 hours, and CD54 (ICAM-1) after 3 hours, compared to an average breakfast. Several other inflammatory genes, like TNFα, MCP1 and CD62l, were up-regulated postprandially, with no differences in response between shakes.

**Table 5:** Changes in expression of genes involved in inflammation in PBMC's after high fat/high energy (HF/HE) or average breakfast control shake consumption.

	Shake	SLR		P-value		
		3h	6h	Shake	Time	Interaction Shake*Time
IL-8	HF/HE	1.23±2.10	1.37±2.56	0.003	<0.001	<0.001
	control	−0.26±2.00	0.68±2.17			
MCP1	HF/HE	0.32±1.20	0.75±1.44	0.049	0.001	0.100
	control	−0.03±1.58	0.28±1.61			
TNFα	HF/HE	0.40±0.38	0.29±0.70	0.101	<0.001	0.213
	control	0.23±0.49	0.22±0.41			
CD62l	HF/HE	0.03±0.39	0.03±0.61	0.517	0.035	0.102
	control	0.02±0.49	0.19 ±0.49			
CD54/	HF/HE	0.25±0.62	−0.03±0.64	0.987	0.001	0.006
ICAM-1	control	0.08±0.70	0.12±0.64			
IL-1β	HF/HE	0.00±0.70	0.00±0.87	0.278	0.216	0.175
	control	−0.27±0.90	−0.05±1.09			
CD11a	HF/HE	−0.10±0.50	−0.13±0.66	0.739	0.690	0.825
	control	−0.08±0.50	−0.08±0.44			

Changes are expressed as signal to log ratio (SLR) compared to baseline values, values are pooled mean ± SD of all three study days (n = 20).

**Figure 2:**
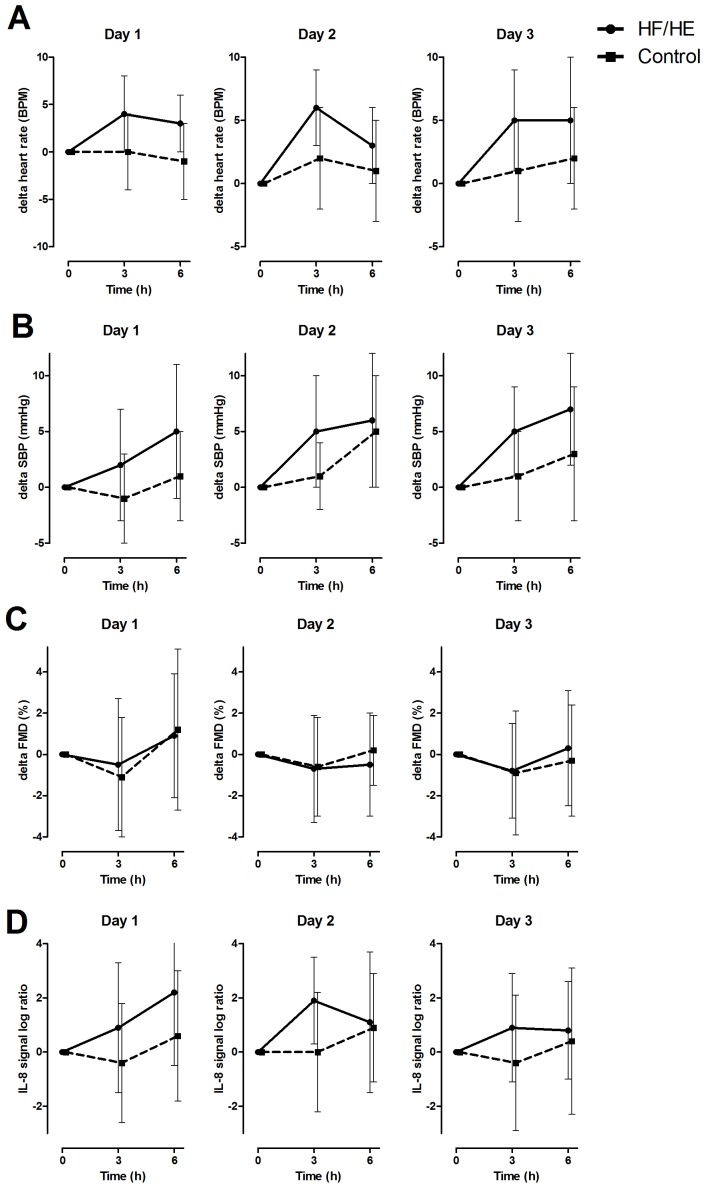
Postprandial changes after consumption of high fat/high energy (HF/HE) or average breakfast shake on heart rate (A), systolic blood pressure (B), FMD (C) and PBMC gene expression of IL-8 (D) subdivided for repeated testing days. Values are mean ± SD.

As illustrated in Figure 2, postprandial responses on HR, SBP, FMD and IL-8 gene expression were similar for each repeated testing day.

## Discussion

In the current study we used a comprehensive approach to study postprandial effects of a HF/HE meal on vascular function and plasma markers of endothelial function and inflammation in young healthy subjects by comparing it to average breakfast milkshake. HF/HE shake consumption was associated with a more pronounced increase in HR, SBP, plasma IL-8 and PBMC gene expression of IL-8 and CD54 (ICAM-1) compared to average breakfast shake consumption.

Only a limited number of studies on postprandial effects of a HF/HE meal on HR and BP have been performed in young healthy individuals. Ayer *et al*., showed an increase in HR 3 hours after HF meal consumption in a healthy young population [9]. However no reference meal was included and it is therefore unclear if the observed effects are caused by HF or by a common postprandial response. With respect to BP, Biston *et al*. observed that ingestion of a mixed meal was associated with a postprandial increase in SBP in young healthy subjects, but they did not compared this response with other meal types [21]. Our observed postprandial increase in BP and HR after both shakes may point towards sympathetic activation after meal intake [22]. Another factor known to affect BP is insulin. By stimulating vasodilatation, insulin is able to reduce BP [23]. The lower BP increase after the average breakfast shake may be due to the higher postprandial insulin concentrations after this shake. However, the insulin peak was increased one hour postprandially, whereas BP was measured three and six hours postprandially.

Although no differences in response between shakes were observed on most vascular measurements, several measures were altered after consumption of both shakes and are therefore caused by consumption of the meal and/or circadian effects. One example is the small but significant postprandial reduction in FMD%. While several previous studies observed a postprandial decrease in FMD after HF consumption, many others found no effect or found an effect with all intervention meals (reviewed by Jackson *et al*.) [5]. Interestingly, many of the studies with no effect were conducted in healthy individuals [9], [24], [25]. We hypothesize that young healthy individuals are still able to handle a high fat and high energy load in such a way that a similar postprandial endothelial response is observed after an averagely consumed breakfast meal.

Besides a postprandial reduction in FMD, consumption of both shakes also altered the AIX and micro-vascular blood flow. The postprandial decrease in AIX is in line with previous findings [9], [10], [26]. The mechanism behind this postprandial reduction in AIX has been less well studied, but arterial smooth muscle relaxation in the general circulation in response to nutrient delivery might be an explanation [26]. AIX values were already negative at baseline and an augmented pressure was therefore not present in this study population. The postprandial decrease in AIX did therefore not affect postprandial central pulse pressure outcomes. The blunted vasoactive compound-induced increase in micro-vascular blood flow after consumption of both shakes was observed for both SNP and Ach and is therefore not endothelium dependent. However, vasoactive compounds were repeatedly administrated at the same location within a few hours. We therefore cannot exclude a reduced sensitivity of the vasculature for these compounds, explaining the observed blunted increase in blood flow.

HF/HE shake consumption resulted in an increase in plasma concentrations and PBMC gene expression of IL-8 compared to the average breakfast shake. Postprandial studies on IL-8 measures are limited. Esposito *et al*. found that serum IL-8 concentrations did not change significantly 4 hours after HF consumption in 30 middle aged diabetic and 30 non-diabetic subjects [27]. However, their HF intervention meal was a mixed meal, which contained besides 52g of fat also 58g of carbohydrates, which is closely to the 49.5g carbohydrates in our average breakfast shake that showed no response on IL-8. Another study that measured plasma concentrations of IL-8 in 8 healthy young men found a non-significant increase two hours after HF meal consumption [14]. However, they did not measure beyond 2 hours postprandially. Although hardly evaluated in postprandial studies, IL-8 is of importance for atherosclerosis development as it is involved in neutrophil activation and recruitment [28] and triggers monocyte adhesion to the vascular endothelium [29]. IL-8 gene expression and production can be regulated by oxidant stress [30], [31]. HF/HE consumption may initiate oxidant stress and thereby trigger endothelial cells to produce and release IL-8, where it is stored in Weibel-Palade bodies [32].

The HF/HE specific decrease in plasma levels of sVCAM-1 is not in line with the general prevailing hypothesis that HF meal consumption is associated with an increase in soluble adhesion molecules [2]. However, postprandial studies regarding soluble adhesion molecules have not shown consistent results. Some studies report elevated sICAM-1 and sVCAM-1 levels after HF meal consumption [33], [34], whereas many others do not [35], [36], [37], [38], [39]. Only one studie found a decrease after HF meal consumption [40]. In general, most studies that did not observe a postprandial increase were performed in younger and often healthy study populations.

Besides the prevailing hypothesis that triglycerides rich particles may increase inflammation, recent human intervention studies demonstrated that high fat/high energy intake can increase circulating endotoxins [41]. These postprandial endotoxins are transported through the gut wall during chylomicron uptake and are able to activate inflammation [42]. Harte *et al*. showed that circulating endotoxins levels show more dramatic postprandial changes in groups with a higher metabolic risk [41]. This might additional explain why in our young healthy study population, several plasma inflammatory cytokines were not altered after HF/HE consumption. To draw a parallel with the measures of vascular function, plasma cytokines were measured at baseline and 3 and 6 hours after shake consumption. As a consequence changes in plasma cytokines that occurred before 3 hours are not detected. It has for example been shown that the cytokines CCL5/RANTES and MCP1 are already changed 1 hour after high fat meal intake [43].

The design used in the current study, with three repeated observations of the intervention within the same individual, allowed to detect small but significant effect sizes, even for measures known for their large variation in reproducibility, such as FMD. In addition, many other previous postprandial studies with high fat challenges used water, fasting or other type of macronutrients as a reference meal. These studies cannot rule out that observed effects are also elicited by a common meal. To enable distinguishing between a HF/HE and a common meal response, we used an averagely consumed breakfast shake as a control. As a consequence, both shakes were not isocaloric and observed differences in response between shakes cannot solely be described to the high fat content but can also be caused by the high energy content or difference in macronutrient composition. Nevertheless, as individuals are daily exposed to a breakfast, the usage of an average breakfast meal as a control mirrors the real life situation.

## Conclusions

Whereas no difference in postprandial response were observed on classical markers of endothelial function, we did observe differences between consumption of a HF/HE and an average breakfast meal on blood pressure and IL-8 in young healthy volunteers. The postprandial increase in blood pressure and plasma IL-8 concentrations after a high fat meal might create a potential harmful environment for the endothelium which in young healthy individuals may not directly affect measures of vascular function, but repeated exposure may on the long run induce endothelial dysfunction. This may be a likely occurrence, since in the western world high fat and high energy meals are regularly consumed. Since IL-8 was one off the only factors different in response between the shakes, it might play a role in dealing with high fat challenges and one of the first factors that may reflect endothelial stress, a stage preceding endothelial dysfunction.

## Supporting Information

Checklist S1 **CONSORT checklist.**
(DOC)

Protocol S1 **Trial protocol.**
(DOC)
