# Supplementary material for: Vascular and Inflammatory High Fat Meal Responses in Young Healthy Men; A Discriminative Role of IL-8 Observed in a Randomized Trial
Source: PLoS One. 2013 Feb 6;8(2):e53474. doi: 10.1371/journal.pone.0053474 (PMC3566159; doi:10.1371/journal.pone.0053474)
Supplement: Protocol S1 — Trial protocol. (DOC) [file pone.0053474.s002.doc]

**Elucidation and monitoring of postprandial endothelial function study**

Diederik Esser, M.Sc.

Lydia A. Afman, Ph.D.

Nutrtion, Metabolism and Genomics Group

Division of Human Nutrition

Wageningen University

Bomenweg 2

6703 HD Wageningen

The Netherlands

e-mail: diederik.esser@wur.nl

Phone: +31 (0)317-480405

Fax: +31 (0)317-48 33 42

**Elucidation and monitoring of postprandial endothelial function study**

| **Protocol ID** | **ABR# 23651** |
| --- | --- |
| **Short title** | **EMPEF** |
| **Version** | **2** |
| **Date** | **08-08-2008** |
| **Principal Investigator** | **Lydia A. Afman, Ph.D.**  **Nutrition, Metabolism and Genomics Group**  **Division of Human Nutrition**  **e-mail: Lydia.Afman@wur.nl**  **phone:+ 31(0)317-485789**  **fax: +31(0)317-483342** |
| **Investigator 2:** | **Diederik Esser, MSc**  **Nutrition, Metabolism and Genomics Group**  **Division of Human Nutrition**  **e-mail: Diederik.Esser@wur.nl**  **phone:+ 31(0)317-480405**  **Fax: +31 (0)317-483342** |
| **Sponsor** | **TI Food and Nutrition** |
| **Independent physician(s)** | **Dr. Marco Mensink (Md, PhD)** |
| **Laboratory sites** | **Division of Human Nutrition**  **Wageningen University**  **Bomenweg 2**  **6703 HD Wageningen**  **The Netherlands** |

**PROTOCOL SIGNATURE SHEET**

| **Name** | **Signature** | **Date** |
| --- | --- | --- |
| **Project leader:**  Prof.dr. M. Müller  **Chair in Nutrition, Metabolism and Genomics** |  |  |
| **Principal investigator:**  Dr.ir. L.A. Afman  **Assistent Professor** |  |  |
| **Investigator 2:**  D.Esser MSc.  **PhD student** |  |  |

TABLE OF CONTENTS

LIST OF ABBREVIATIONS AND RELEVANT DEFINITIONS [6](#__RefHeading___Toc201372055)

SUMMARY [7](#__RefHeading___Toc201372056)

1. INTRODUCTION AND RATIONALE [8](#__RefHeading___Toc201372057)

1.1 Scientific relevance [8](#__RefHeading___Toc201372058)

1.2 Functional measures of endothelial function [8](#__RefHeading___Toc201372059)

1.3 Elucidation of how a high-fat meal can induce endothelial dysfunction [9](#__RefHeading___Toc201372060)

1.4 Study aim [9](#__RefHeading___Toc201372061)

2. OBJECTIVES [10](#__RefHeading___Toc201372062)

2.1 Research questions and hypothesis [10](#__RefHeading___Toc201372063)

3. STUDY DESIGN [11](#__RefHeading___Toc201372064)

3.1 Intervention study [11](#__RefHeading___Toc201372065)

4. STUDY POPULATION [11](#__RefHeading___Toc201372066)

4.1 Population [11](#__RefHeading___Toc201372067)

4.2 Screening visit [11](#__RefHeading___Toc201372068)

4.3 Exclusion criteria [12](#__RefHeading___Toc201372069)

4.4 Sample size calculation [13](#__RefHeading___Toc201372070)

5. INTERVENTION [14](#__RefHeading___Toc201372071)

5.1 Intervention meals [14](#__RefHeading___Toc201372072)

5.2 Guidelines during the study period [14](#__RefHeading___Toc201372073)

6. METHODS [15](#__RefHeading___Toc201372074)

6.1 Study outcomes [15](#__RefHeading___Toc201372075)

*6.1.1 Measurements of primary study outcomes* [15](#__RefHeading___Toc201372076)

*6.1.2 Measurements of secondary study outcomes* [16](#__RefHeading___Toc201372077)

*6.1.3 Measurements of tertiary study outcomes* [17](#__RefHeading___Toc201372078)

6.2 Study procedures [18](#__RefHeading___Toc201372079)

6.2.1 Information meeting [18](#__RefHeading___Toc201372080)

6.2.2 Screening [18](#__RefHeading___Toc201372081)

6.2.3 Preparations prior to the Intervention [18](#__RefHeading___Toc201372082)

6.3 Withdrawal of individual participants [22](#__RefHeading___Toc201372083)

6.4 Follow-up of participants after withdrawal [22](#__RefHeading___Toc201372084)

6.5 Premature termination of the study [22](#__RefHeading___Toc201372085)

7. SAFETY REPORTING [23](#__RefHeading___Toc201372086)

7.1 Section 10 WMO event [23](#__RefHeading___Toc201372087)

7.2 Adverse and serious adverse events [23](#__RefHeading___Toc201372088)

7.3 Follow-up of adverse events [23](#__RefHeading___Toc201372089)

8. STATISTICAL ANALYSIS [24](#__RefHeading___Toc201372090)

9. ETHICAL CONSIDERATIONS [25](#__RefHeading___Toc201372091)

9.1 Regulation statement [25](#__RefHeading___Toc201372092)

9.2 Recruitment and consent [25](#__RefHeading___Toc201372093)

9.3 Privacy [25](#__RefHeading___Toc201372094)

9.4 Benefits and risks assessment, group relatedness [25](#__RefHeading___Toc201372095)

9.4.1 Potential value of the research [25](#__RefHeading___Toc201372096)

9.4.2 Benefits for the participants [25](#__RefHeading___Toc201372097)

9.4.3 Risks for the participants [26](#__RefHeading___Toc201372098)

9.5 Compensation for injury [27](#__RefHeading___Toc201372099)

10. ADMINISTRATIVE ASPECTS AND PUBLICATION [28](#__RefHeading___Toc201372100)

10.1 Handling and storage of data and documents [28](#__RefHeading___Toc201372101)

10.2 Amendments [28](#__RefHeading___Toc201372102)

10.3 End of study report [28](#__RefHeading___Toc201372103)

10.4 Public disclosure and publication policy [28](#__RefHeading___Toc201372104)

11. REFERENCES [29](#__RefHeading___Toc201372105)

**LIST OF ABBREVIATIONS AND RELEVANT DEFINITIONS**

Ach Acetylcholine

AIx Augmentation index

BMI Body mass index

BP Blood pressure

CAM Cellular adhesion molecules

CC Compliance coefficient

CCMO Central Committee on research Involving Human Subjects

CRP C-reactive protein

DC Distensibility coefficient

ED Endothelial dysfunction

EF Endothelial function

(F)FA (Free) Fatty acids

FMD Flow-mediated dilatation

Hb Hemoglobin

HF High-fat

IC Informed consent

IMP Investigational Medicinal Product

IMT Intima-media complex thickness

METC Medical ethical commission (in Dutch: medisch ethische commissie)

PBMC Peripheral blood mononuclear cells

Pwv Pulse wave velocity

Q-PCR Quantitative real time polymerase chain reaction

(S)AE (Serious) Adverse Event

SFA Saturated fatty acids

Sponsor The sponsor is the party that commissions the organization or performance of the research, for example a pharmaceutical company, academic hospital, scientific organization or investigator. A party that provides funding for a study but does not commission it is not regarded as the sponsor, but referred to as a subsidizing party

TAG Triacylglycerol

vWF Von Willebrand factor

WMO Medical Research Involving Human Subjects Act (In Dutch: Wet Medisch-Wetenschappelijk Onderzoek met Mensen)

YEM Young’s elastic modulus

**SUMMARY**

**Rationale:**

Endothelial dysfunction (ED) is a hallmark for the initial stage of vascular dysfunction and has been associated with diet-related disorders such as cardiovascular disease. This makes prevention of ED an important health target. From previous studies we know that a high-fat (HF) meal (challenge) impairs postprandial endothelial function (EF). Current studies only evaluated the effect of a HF meal on Flow Mediated Dilatation (FMD), a measure of macro vascular EF. This methodology (FMD) however, is time consuming and large variations in reproducibility are reported in literature. The question remains whether other types of macro- and micro vascular EF measurements can be used to observe ED after a HF meal that are more accurate, faster and easier to perform. In addition, it is know that the postprandial phase results in activation of leukocytes. This activation of leukocytes is likely to contribute to ED, but the exact underling mechanism remains unclear.

**Objectives:** The primary objectives are formulated, to evaluate the effect of a HF meal on EF on both, macro- and micro vascular level by evaluating different measures for EF. This will be performed with the ultimate goal to establish a reliable model to evaluate HF meal induced ED, which can be used in further studies. Our secondary objectives are to elucidate the underling mechanisms of HF meal-induced ED and to identify early biomarkers of ED.

**Study design and study population:** Double-blind cross-over intervention with 20 healthy Caucasian men (age 18-30, 18<BMI<25)

**Intervention:** Each participant will consume a HF milkshake or a ‘control’ milkshake (comparable with a normal breakfast) after overnight fasting.

**Study outcome**: The primary study outcomes are the functional measurements on macro-and micro vascular function before and 3 and 6 hours after a HF meal. Our secondary study outcomes are peripheral blood mononuclear cells (PBMC) gene expression profiles, white blood cell activation markers, plasma cytokine profiles and other plasma markers of endothelial function before and after a HF challenge. These findings will thereafter be correlated with the outcome of the functional measurements.

**Nature and extent of the burden and risks, benefit and group relatedness:** During a screening visit 3 ml blood will be drawn and urine will be collected after a overnight fast and body weight and length will be measured. A general and medical questionnaire will be used. During the study each participant will consume a milkshake containing 95 grams of fat or a ‘control’ milkshake after a overnight fast. Both interventions will be performed in triplicates and all 6 testing days are randomly divided over a 15 week period. Functional measurements of ED will be performed at baseline and 3 and 6 hours after the intervention. At baseline (-1.5 hours) and 1,2,3,5 and 6 hours after the intervention, blood will be collected by using a venflon. The total amount of blood will be 192 ml per day. Hb values of each participant will be monitored during the study to be sure that blood collection will not lead to anaemia. The time investment requested from the participants is 1.5 hours at the information meeting, 0.5 hours at a screening session, and 6 x 9 hours during the intervention days. The risks associated with venous blood drawing using the venflon technique are minimal. The consumption of the milkshake is not expected to be associated with discomfort and all performed functional measurements of ED are non-invasive and risks are therefore minimal.

**1. INTRODUCTION AND RATIONALE**

**1.1 Scientific relevance**

The endothelium is a thin layer of cells that lines the interior surface of the blood vessels. The endothelium responds to changes in the physical, metabolic, and inflammatory environment and by doing this, it maintains vascular homeostasis in the arteries and in the microcirculation. Endothelial dysfunction (ED) is a hallmark for the initial stage of vascular dysfunction^1^ and has been associated with several diet-related diseases^2^. As nicely reviewed by Khazaei *et al.* ^3^, several studies support the hypothesis that reduction of endothelial dysfunction may lead to reduced cardiovascular morbidity and mortality (e.g. heart disease, stroke). This makes prevention of ED an important health target.

From previous studies we know that nutritional challenges result in an impaired postprandial endothelial function (EF) as measured by flow-mediated dilatation (FMD), markers of inflammation and markers of oxidative stress^4-9^. These effects are reversible and temporally. A high-fat (HF) challenge, with a fat content between 80-100g, has the strongest and most clear effect on EF compared to other nutritional challenges^4, 10^.

**1.2 Functional measures of endothelial function**

EF can be assessed by several methodologies/functional measurements of which each has its own advantages and drawbacks. Currently, HF meal induced ED is only studied on a macro vascular level by using FMD. Flow mediated dilatation is a commonly used technique to investigate the ability of vascular endothelial cells to respond to changes in shear stress. However, this technique is time consuming and large variations in reproducibility are found in the literature (reviewed by De Roos *et al*. 2003 ^11^).

There are other methodologies to monitor EF besides FMD, although these are not applied to monitor HF meal induced ED. Another way to (indirectly) indicate EF on macro vascular level is by measuring the arterial stiffness^12^. Arterial stiffness (i.e. impairment of the cushioning capacity) leads to an increase in systolic blood pressure and can be estimated by quantifying pulse pressure, but is better described by distensibility and compliance coefficients, pulse wave velocity and wave reflection. It is also possible to monitor EF on a micro vascular level. Abahji *et al.* (2006)^13^ observed impaired micro vascular EF after acute hyperhomocysteinemia by using ionthophoresis techniques. The question remains if these other methodologies are also applicable to observe a HF meal induced ED.

**1.3 Elucidation of how a high-fat meal can induce endothelial dysfunction**

So far, it is not completely known how a HF meal results in a impaired EF. However, it is known that triglycerides and markers of inflammation are increased during a postprandial response^4, 10^. In addition, van Oostrom *et al.* (2004) observed an increased expression of activation markers on several white blood cells, including neutrophiles and monocytes, after a HF challenge. These activation markers (CD11A, CD11B and CD62L) are known to interact with the endothelium^10^. Postprandial activation of white blood cells may therefore result in an increased adhesive capacity of these cells to the endothelium. This might have an negative effects on EF, since prevention of adherence of leukocytes to the endothelium results in preservation of EF^14^. Evaluating these and other white blood cell activation markers during a HF postprandial response, may give information about of the role of activated blood cells in postprandial ED.

Several factors are likely to be important precursors of ED, but the precise relationship between these factors and ED is still poorly understood and reliable early biomarkers are lacking. In humans, EF is now assessed biochemically by measuring circulating levels of oxidative products and markers of endothelial activation and inflammation. Important markers in this respect are NO, NOx, prostacyclin, cyclic adenosine monophosphate, cyclic guanosine monophosphate, cytokines, endothelin, intercellular adhesion molecule-1 (ICAM-1), vascular cell adhesion molecule-1 (VCAM-1), von Willebrand factor, plasminogen activator inhibitor-1, or thrombomodulin. Information about changes in PBMC gene expression profiles and plasma cytokine profiles during postprandial ED could lead to novel early biomarkers of ED and may provide us with leads for possible mechanisms.

**1.4 Study aim**

In the current study, we aim to evaluate the effect of a HF meal on EF on both, macro- and micro vascular level by evaluating different measures for EF. This will be performed with the ultimate goal to establish a reliable functional measure for HF meal induced ED, which can be used in further studies. Local and regional arterial stiffness and macro vascular blood flow will be evaluated in order to observe an effect on macro-vascular level and micro vascular blood flow will be evaluated to observe an effect on micro-vascular level. Comparison of these measures will allow us to establish the most accurate but also easy and fast method for monitoring HF meal induced ED.

In addition we aim to obtain more knowledge on the underling mechanisms of HF meal-induced ED and to identify early biomarkers for ED. In order to do so we will investigate HF meal-induced changes in gene expression profiles of peripheral blood mononuclear cells (PBMC), activation markers of white blood cells, plasma cytokine profiles and other markers of EF (see paragraph 1.4). These findings will thereafter be correlated with the outcome of the functional measurements to obtain possible leads for biomarkers and mechanism.

**2. OBJECTIVES**

The objectives are formulated to investigate the effect of a HF meal on EF. The primary objectives are to evaluate the effect of a HF meal on several measures of macro- and micro vascular EF. Our secondary objectives are to obtain more knowledge on the underling mechanisms of HF meal-induced ED and to identify early biomarkers of ED.

**2.1 Research questions and hypothesis**

1. Does a HF meal have an effect on EF that can be measured with several measures of micro- and macro-vascular function and which of these methodologies is best applicable to measure HF meal induced ED?

🡪 *Both micro- and macro-vascular endothelial function will be affected after a HF meal. All used methodologies can therefore be used for measuring HF induced ED, although some might be more applicable (more accurate, easier to use).*

1. Does a HF meal results in changes in PBMC gene expression profiles, white blood cell activation markers, plasma cytokine profiles and other markers of ED and can these observed effects be correlated to the functional measurements of ED?

🡪 *A high fat challenge will have an effect on PBMC gene expression profiles, white blood cell activation, plasma cytokine profiles and other plasma markers of ED. Some of these changes will correlated either positively of negatively to the outcomes of the functional measurements and might be good candidate early biomarkers for ED and will provide us with leads for a possible mechanism.*

**3. STUDY DESIGN**

**3.1 Intervention study**

This study is a double-blind cross-over intervention (challenge) study, planned to run (incl. a 2 week break during Christmas/ new year) from 20-10-2008 till 15-02-2009. A total of 20 healthy male individuals will receive a) a milkshake containing 95g of fat (high-fat challenge) or b) a milkshake containing the same nutritional and energetic value of an average breakfast (based on ‘voedselconsumentenpeiling’ 2003 from TNO). Data obtained with this ‘control’ milkshake will not directly be compared to the HF milkshake, but will be used to obtain knowledge about normal circadian rhythms in EF. Both interventions will be performed in triplicates, to obtain information on variation in the different measures of EF and the variation in PBMC gene expression, white blood cell activation markers, plasma cytokine profiles and other plasma markers of ED. This means that each individual will visit the department on 6 different days. All testing days will be randomly divided over the study time period, with a minimal period between the testing days of 7 days, in order to exclude carry over effects. Randomization is performed in which only the variants ABABAB and BABABA are possible. Two participants will be evaluated on each testing day (see paragraph 6.2.4)

**4. STUDY POPULATION**

**4.1 Population**

Healthy Caucasian men (from the student population of Wageningen University), age between 18 and 30 years. Women are excluded from the study population since macrovascular EF, microvascular EF and arterial stiffness outcomes show variation during the menstrual cycle ^15-17^.

**4.2 Screening visit**

Before the screening, volunteers have to give their written informed consent. A general and medical questionnaire will be filled out, containing questions concerning our exclusion criteria. Fasting blood and urine samples will be collected and the following parameters will be determined for all volunteers:

• Fasting blood glucose

• Fasting urinary glucose and protein

• Length and weight

- Blood pressure (BP)
- Hemoglobin (Hb) value

Furthermore, we will look at the suitability of the veins for taking blood samples (for using a venflon) and functional ED measurements. After the screening visit, the participant will receive a breakfast.

**4.3 Exclusion criteria**

- Allergic to cow milk or dairy products
- Body mass index (BMI) < 18 or > 25 kg/m^2^
- Urine glucose concentrations outside normal ranges (>0,25 g/l)
- Fasting blood glucose outside the normal range (3 - 5.5 mmol/L)
- Tobacco smoking
- Taking medication or food supplements.
- Received inoculations within 2 months of starting the study or planned to during the study
- Donated or intended to donate blood from 2 months before the study till two months after the study
- Blood Hb values below 8.4 mmol/L
- Diagnosed with any long-term medical condition (e.g., diabetes, hemophilia, cardiovascular disease, anemia, gastrointestinal disease, renal failure)
- High blood pressure (systolic BP> 140 mmHg and/or diastolic BP>90 mmHg)

**4.4 Sample size calculation**

Based on the methodological paper by de Roos *et al. (2003) ^11^*we can estimate that we need 7 subjects to obtain a 6 FMD% difference before and after the fat load with 90% power and alpha of 5%, using an within-subjects SD of 2.8 FMD%. Van Oostrom *et al.*(2003) observed a reduction from 13 FMD% to 3 FMD% after a high-fat load in healthy men ^4^. However, we know that the within-subject coefficient of variation for FMD can be large and that FMD measures differ between studies. ^13^ The latter may be due to methodological reasons^18^. Hence we propose to include 20 subjects, which enables us to detect a difference in 3 units of endothelial function as measured with ionthophoresis (paired t-test, SD 4.5 units, power 80%, alpha 5%), which seems plausible ^13^.

**5. INTERVENTION**

**5.1 Intervention meals**

Each participant will receive a high-fat challenge or a ‘control’ meal. The HF meal will consist of a milkshake, containing a total amount of 95g of fat (consisting of mainly saturated fatty acids) and the total volume that will be consumed is 500ml. The ‘control’ meal will consist of a milkshake of witch the content can be compared to a normal breakfast. Both shakes/meals should be consumed within 10min. Table 1 describes the ingredients, macronutrient composition and total energy intake for each shake/meal.

**Table 1:** *A)* *Ingredients used for both milkshakes and B) Nutritional values of the two milkshakes according to Nevo2006.*

A)

| HF milkshake | ‘control’ milkshake |
| --- | --- |
| 53% Whipped cream  3% Sugar  44% Water | 42% full cream milk  47% full cream yoghurt  10% lemonade  1% wheat fiber |

**B)**

|  | HF milkshake (500ml) | ‘control’ milkshake (500ml) |
| --- | --- | --- |
| Energy (KJ) | 3992.08 | 1674.00 |
| Protein (g) | 6.13 | 17.00 |
| Fat (g)  Of which saturated (g) | 94.83  54.22 | 14.50  9.00 |
| Carbohydrates (g) | 22.31 | 49.70 |
| fiber (g) | 0 | 2.30 |

**5.2 Guidelines during the study period**

All participants will be advised to keep the same dietary habits as before the study period. In order to standardize the participants‘ nutritional status before each testing day, the participants will be asked to:

- Consume a low fat evening meal (<10g) the day before each study day (will be provided).
- Not consume any alcohol the day before each study day
- Avoid strenuous exercise the day before each study day
- Fast overnight before each study day (min of 10 hours).

Details on how to live up to these guidelines will be given (and discussed) to each participant before the start of the study.

**6. METHODS**

**6.1 Study outcomes**

***6.1.1 Measurements of primary study outcomes***

Four different non invasive functional measurements will be used to establish a reliable method to measure HF induced ED and to observe the effect of a HF meal on systemic arterial stiffness, local arterial stiffness, macro circulation and micro circulation. The total protocol of the EF measurements will take 1.5 hours (for details see paragraph 6.2.4). Functional measurements will be performed at baseline (T=-1.5) and 3 and 6 hours after the challenge, because previous studies observed a maximal response in macro vascular circulation by FMD after 3 hours that returned to normal after 6 hours^4, 9^. All functional measurements will be performed on the right side of the body in a climate controlled room.

**Macro vascular regional arterial stiffness by tonometry**

Pressure sensors (applanation tonometers) are applied at the carotid and femoral artery to record pressure waveforms (reviewed by Stehouwer *et al*.^19^). From these waveforms we can conduct two measurements of arterial stiffness: the pulse wave velocity (pwv) and the augmentation index (AIx).

- The pulse wave velocity (pwv): The speed of the pressure wave travelling along an arterial segment (e.g. carotid-femoral). It is determined by the length of the arterial segment divided by the time it takes for a pressure wave to travel from the carotid artery to the femoral artery. An ECG is necessary as a reference. Greater pwv values indicate greater stiffness.
- Augmentation index (AIx): The supplementary increase in blood pressure during systole, due to the reflection of the forward travelling pressure waves from the peripheral circulation. AIx is an estimate of stiffness and wave reflection and therefore provides indirect information on arterial stiffness.

**Macro vascular local arterial stiffness by ultrasound**

Ultra-sonography (echo tracking) of the carotid arterial wall (reviewed by Stehouwer *et al*.^19^) provides several estimates:

- Compliance coefficient (CC): Absolute change in lumen area for given increase in pressure.
- Distensibility coefficient (DC): Relative change in lumen area for a given change in pressure
- Young’s elastic modulus (YEM): The pressure step per squared centimeter required for (a theoretical) 100% stretch from resting length, determined by the arterial diameter (D), divided by DC, multiplied by the thickness of the intima-media complex (IMT): YEM = D/DCxIMT.

These estimates are determined directly from simultaneous changes in (local) pulse pressure and arterial diameter or area. The lower the DC or CC and the higher the YEM of an artery the higher is its stiffness. Local arterial stiffness will also be determined for the brachial artery since all above mentioned estimates will also be determined during the measurements for FMD (see below).

**Macro vascular circulation by flow-mediated dilatation (FMD)**

The FMD will be assessed by ultra-sonography (echo tracking) of the brachial artery and is based on the ability of vascular endothelial cells to respond to changes in shear stress. FMD is estimated as the percentage increase in vessel diameter from baseline conditions to maximum vessel diameter during hyperemia.

**Micro vascular circulation by Ionthophoresis**

Small amounts of a ionic vaso-active drug will diffuse through the skin by an electric field. An endothelial dependent (acetylcholine) and an endothelial non-dependent (sodium nitroprusside) vaso-active drug will be used to perfuse the skin and changes in skin blood flow will be assessed by Laser Doppler monitoring/ imaging.

***6.1.2 Measurements of secondary study outcomes***

Blood samples will be taken from the left arm, which is opposite to the one used for measuring functional ED measures at baseline (-1.5h) and at 1, 2, 3, 5, 6 hours after the milkshake by using a venflon.

**PBMC gene expression profiles** will be determined by using whole genome NuGO Affymetrix microarrays at baseline (-1.5h) and 3 and 6 hours after receiving the challenge. Expression profiles of genes selected from the microarrays will be assessed at all other blood collection time points by quantitative real time PCR (Q-PCR)

**White blood cell activation markers** will be determined at baseline and after 3 and 6 hours by using flow cytometry analysis (coulter Epics XL.MCL flow cytometry by Beckman Coulter)

**Plasma cytokine profiles** will be tested by quantitative immunoassays at rules Based Medicine laboratories (Austin, TX) at all blood collection time points. The cytokine profile of a panel of 120 proteins including will be determined. For detail on the 120 proteins, see:

<http://www.rulesbasedmedicine.com/services_human_antigen.asp>.

**Known plasma markers of endothelial function such as** NO, NOx, prostacyclin, cyclic adenosine monophosphate, cyclic guanosine monophosphate, cytokines, endothelin, intercellular adhesion molecule-1, vascular cell adhesion molecule-1, von Willebrand factor, plasminogen activator inhibitor-1, or thrombomodulin, selectins, integrins, HDL functionality, HDL lipid mediators and extracellular phospholipase secretory phospholipase A2-IIA will be determined at baseline and after 3 and 6 hours. Markers that are changed at these time points will also be measured at all other blood collection time points by using quantitative immunoassays, ELISA and at rules Based Medicine laboratories (see above).

**6.1.3 *Measurements of tertiary (additional) study outcomes***

Plasma glucose, triacylglycerol (TAG), FFA and insulin concentrations will be determined at the biochemical laboratory in Velp. Outcomes of these parameters are known and provide information about the normal physiological response. These tertiary study outcomes will therefore be used as a control.

**6.2 Study procedures**

**6.2.1 Information meeting**

An information meeting will be organized 6 weeks prior of the study. In this meeting the investigators will explain the background, objectives and set up of the study. Also the possible risks and burdens will be explained and during this meeting there will be time for the potential participants to ask questions.

**6.2.2 Screening**

There will be a screening visit planned 4 weeks prior of the beginning of the study in order to recruit participants who fulfill all criteria. The following data will be collected:

- Written informed consent
- 3ml of blood for fasting blood glucose
- Fasting urinary glucose and protein concentrations
- Length and height
- Hb values
- Blood pressure values
- A general medical questionnaire will be filled in

**6.2.3 Preparations prior to the Intervention**

All participants will receive a diary 2 weeks prior to the study. In this diary, the participants can keep track of any signs of illness, any deviations from the protocol or any possible side effects. In addition, further details (e.g. logistical) of the experiment will be elucidated.

**6.2.4 Intervention**

Participants will be asked to check in 30 min prior to the first data collection point (T=-2h) in order to be sure that measurements are taken at a ‘resting’ state. Before the start of each study day, the Hb value of each participant will be determined by using a hemoglobin analyzer (HemoCue 201). Participants with a Hb value below 8.4 mmol/L will not be measured and are asked to make a new appointment. If Hb values are normal (e.g. above 8.4mmol/L), a venflon will be placed into the antecubital vein of the participant’s forearm by qualified and experienced personnel. Functional measurements will be performed at baseline (T= -1.5h) and 3 and 6 hours after the intervention. The total time required for the functional measurements is set at 1.5 hours in total and the protocol is as followed:

- 15 min prior to the measurements, participants are asked to lie down on the bed
- Blood pressure will be measured (required for some measures below)
- Macro vascular regional arterial stiffness will be assessed by tonometry
- Macro vascular local carotid arterial stiffness will be assessed by ultrasound
- Macro vascular blood circulation will be assessed by FMD/ ultrasound
- Micro vascular blood circulation will be assessed by iontophoresis

Blood will be collected at baseline (T=-1.5) and 1, 2, 3, 5, and 6 hours after the intervention. An overview of the data collection points on each study day is shown in table 2.

**Table 2:** *protocol on each study day. NB. Data will be collected for two participants each day. The second participant will receive the same protocol, only all time points are delayed by one and a half hour.*

| Intervention time, after overnight fasting (in hours (h)) | -1.5h | 0h | 1h | 2h | 3h | 5h | 6h |
| --- | --- | --- | --- | --- | --- | --- | --- |
|  | (7.30am) | (9.00am) | (10.00am) | (11.00am) | (12.00am) | (14.00am) | (15.00am) |
| Consumption of the test meal |  | X |  |  |  |  |  |
| **Primary measurements** |  |  |  |  |  |  |  |
| Macro vascular:  Arterial stiffness  by tonometry | X |  |  |  | X |  | X |
| by ultrasound | X |  |  |  | X |  | X |
| Flow mediated dilatation | X |  |  |  | X |  | X |
| Micro vascular:  Iothophoresis | X |  |  |  | X |  | X |
| **Secondary measurements** |  |  |  |  |  |  |  |
| Total blood sampling (ml) | 56 |  | 8 | 8 | 56 | 8 | 56 |
| PBMC gene expression |  |  |  |  |  |  |  |
| By microarray analysis | X |  |  |  | X |  | X |
| By Q-PCR | X |  | X | X | X | X | X |
| White blood cell activation | X |  |  |  | X |  | X |
| Cytokines and markers of ED | X |  | X | X | X | X | X |
| **Tertiary measurements** |  |  |  |  |  |  |  |
| Plasma Insulin/FFA/TAG/glucose | X |  | X | X | X | X | X |

A total volume of 192ml will be collected from each participant during each collection day. An elucidation of this volume is given in table 3.

***Table 3:*** *Elucidation of blood volumes (ml)* taken for the secondary measurements

|  | T=-1.5 | T=1 | T=2 | T=3 | T=5 | T=6 | Overall |
| --- | --- | --- | --- | --- | --- | --- | --- |
| PBMC gene expression profiles | 16 | 4 | 4 | 16 | 4 | 16 |  |
| White blood cell activation markers | 20 | - | - | 20 | - | 20 |  |
| Cytokine and other ED markers | 20 | 4 | 4 | 20 | 4 | 20 |  |
| Total | 56 | 8 | 8 | 56 | 8 | 56 | 192 |

The participants will spend the entire day (approximately 9 hours) in the research room, where they will be able to read books, watch television/DVD, or relax in comfortable chairs. During the whole experiment , at least 2 persons other than the participants are in the room. At the end of the study day the participants will be offered a warm meal . At the end of the study, diaries will be collected.

A total of two participants will be evaluated on a single testing day. Person A will start at 7.00h and will finish at 16.30h. Person B will start at 8.30h and will finish at 18.00h. An overview of a single testing day in which two participants will be evaluated is given in table 4.

**Table 4:** *Time scheme during a study day. Two participants will be measured each study day. Person A will start at 7.00h and will finish at 16.30h. Person B will start at 8.30h and will finish at 18.00h. Time points in which blood will be collected are marked with a *.*

| **Time** | **T** | **Person A** | **Person B** | **T** | **Time** |
| --- | --- | --- | --- | --- | --- |
| **7.00** | -2 | arrival subject |  |  | **7.00** |
| 7.15 |  |  |  |  | 7.15 |
| 7.30 | -1.5 * | Functional |  |  | 7.30 |
| 7.45 |  | measurements |  |  | 7.45 |
| **8.00** | -1 | baseline |  |  | **8.00** |
| 8.15 |  |  |  |  | 8.15 |
| 8.30 |  |  | arrival subject | -2 | 8.30 |
| 8.45 |  |  |  |  | 8.45 |
| **9.00** | 0 | **Challenge** | Functional | -1.5* | **9.00** |
| 9.15 |  |  | measurements |  | 9.15 |
| 9.30 |  |  | baseline | -1 | 9.30 |
| 9.45 |  |  |  |  | 9.45 |
| **10.00** | 1* |  |  |  | **10.00** |
| 10.15 |  |  |  |  | 10.15 |
| 10.30 |  |  | **Challenge** | 0 | 10.30 |
| 10.45 |  |  |  |  | 10.45 |
| **11.00** | 2* |  |  |  | **11.00** |
| 11.15 |  |  |  |  | 11.15 |
| 11.30 |  |  |  | 1* | 11.30 |
| 11.45 |  |  |  |  | 11.45 |
| **12.00** | 3* | Functional |  |  | **12.00** |
| 12.15 |  | measurements |  |  | 12.15 |
| 12.30 |  | T=3 |  | 2* | 12.30 |
| 12.45 |  |  |  |  | 12.45 |
| **13.00** | 4 |  |  |  | **13.00** |
| 13.15 |  |  |  |  | 13.15 |
| 13.30 |  |  | Functional | 3* | 13.30 |
| 13.45 |  |  | measurements |  | 13.45 |
| **14.00** | 5* |  | T=3 |  | **14.00** |
| 14.15 |  |  |  |  | 14.15 |
| 14.30 |  |  |  | 4 | 14.30 |
| 14.45 |  |  |  |  | 14.45 |
| **15.00** | 6* | Functional |  |  | **15.00** |
| 15.15 |  | measurements |  |  | 15.15 |
| 15.30 |  | T=6 |  | 5* | 15.30 |
| 15.45 |  |  |  |  | 15.45 |
| **16.00** | 7 |  |  |  | **16.00** |
| 16.15 |  |  |  |  | 16.15 |
| 16.30 |  |  | Functional | 6* | 16.30 |
| 16.45 |  |  | measurements |  | 16.45 |
| **17.00** |  |  | T=6 |  | **17.00** |
| 17.15 |  |  |  |  | 17.15 |
| 17.30 |  |  |  | 7 | 17.30 |
| 17.45 |  |  |  |  | 17.45 |

**6.3 Withdrawal of individual participants**

Participants can leave the study at any time for any reason if they wish to do so without any consequences. The investigators can decide to withdraw a participant from the study for urgent medical reasons. After withdrawal of a participant, they will be replaced if this is still compatible with the study guidelines

**6.4 Follow-up of participants after withdrawal**

After possible withdrawal, no follow-up of participants will take place. In case of withdrawal due to medical complications, participants will be referred to a general physician.

**6.5 Premature termination of the study**

The procedures for premature termination of the study can be found in the adverse events protocol of the division Human Nutrition of Wageningen University.

**7. SAFETY REPORTING**

**7.1 Section 10 WMO event**

When it appears that the disadvantages of participation may be significant greater than was foreseen in the research protocol, participants and the reviewing accredited METC will be informed according to section 10, subsection 1, of the WMO.

**7.2 Adverse and serious adverse events**

Adverse events (AE) include any undesirable experience occurring to a participant during a clinical trial, whether or not considered related to the investigation. All AE reported by the participant or observed by the investigators will be recorded.

A serious adverse event (SAE) is any untoward medical occurrence or effect that at any dose results in death:

- Is life threatening (at the time of the event)
- Requires hospitalization or prolongation of hospitalization.
- Results in persistent or significant disabilities or incapacity
- Is a congenital anomaly
- Is a new event of the trial likely to affect the safety of the participants, such as an unexpected outcome of an adverse reaction, lack of efficacy of an IMP used for the treatment of a life threatening disease, major safety finding from a newly completed animal study, etc.

All SAE will be reported to the accredited METC that approved the protocol, according to the requirements of that METC.

**7.3 Follow-up of adverse events**

All AE will be followed until they have abated, or a stable situation has been reached. Depending on the event, follow-up may require additional test or medical procedures as indicated, and/or referral to the general physician or a medical specialist.

**8. STATISTICAL ANALYSIS**

To observe an effect of HF on EF, changes in primary outcome variables will be tested using repeated measures ANOVA (mixed model) using pre-defined contrasts, i.e. 3 versus 0 hrs and 6 versus 3 hrs. ANOVA will also be used to quantify the within- and between subject variation of the various measurements.

Changes in secondary outcome variables will be tested using repeated measures ANOVA (mixed model) using pre-defined contrasts, i.e. 3 versus 0 hrs and 6 versus 3 hrs. Additionally, incremental areas under the curve will be calculated for all significant altered secondary outcome variables using the trapezoïdal rule and compared between meals using one-way ANOVA. All statistical analysis will be performed with SPSS version 15.0.

Data analysis of the microarrays will be performed using a Bayesian linear regression model (implemented in the software package LIMMA; [www.bioconductor.org](http://www.bioconductor.org/)). Raw microarray data will be normalized using RMA quantile normalization. Statistically significant changed genes will be identified by student paired t-test followed by a false discovery rate correction (FDR) The latter is used to correct for multiple testing.

**9. ETHICAL CONSIDERATIONS**

**9.1 Regulation statement**

The study will be conducted according to the principles of the Declaration of Helsinki (Tokyo, 2004) and in accordance with the Medical Research Involving Human Subjects Act (WMO).

**9.2 Recruitment and consent**

Recruitment will take place at the campus of Wageningen University, by means of posters and though the mailing list for potential study participants of the division of human nutrition of Wageningen University. No directly-related colleagues will be recruited for this study. An information booklet will be provided during the information meeting organized 6 weeks before the start of the study. Written informed consent will be obtained during a screening visit 4 weeks before the start of the study. In the informed consent we will ask the participants if they want to give permission for anonymous storage of their blood samples for possible additional analyses in the future. We will ask the METC for approval before performing additional analyses.

**9.3 Privacy**

Personal data will be stored in a closed locker and a password-protected file, to which only the investigators have access. Samples will be coded and destroyed within twelve years. Only the investigators have access to the code.

**9.4 Benefits and risks assessment, group relatedness**

**9.4.1 Potential value of the research**

This study evaluates the effect of a HF meal on macro- and micro vascular EF. With these outcomes, we will be able to indicate the accuracy of the used methods in assessment of postprandial ED. The most reliable, accurate and easy method will be used in further diet related ED experiments. Gene expression profiles of PBMC’s, activation markers of white blood cells, plasma cytokine profiles and other plasma markers of ED could provide valuable knowledge about the molecular mechanisms behind ED especially when compared with corresponding functional measurement data.

**9.4.2 Benefits for the participants**

Participants that complete the study will receive €300.00 Participants are free to withdraw from further participation for any reason and at any time during the trial and will receive a proportional bonus for the effort they made (50 euro for each completed testing day). Participants can receive a summary of the results with averages of the group after the end of the study. Furthermore, participants will receive a warm meal the day before each study day and at the end of each study day.

**9.4.3 Risks for the participants**

There are minor risks for the participants during the intervention. The consumption of the HF shakes is not expected to be associated with any risk, although in rare cases it could lead to mild gastro-intestinal discomfort. Participants also can experience a fatty taste in their mouth after intake of the HF milkshake. No discomfort is expected with the consumption of the ‘control’ milkshake since it correlates to a normal breakfast.

Before each study day, the Hb value of each participant will be determined using a HemoCue 201 (provided by Sanquin/ blood bank). Participants will not be measured if this value is below 8.4 mmol/L. Blood collection will therefore not lead to anemia. Venapunctures/insertion of the venflon can occasionally cause a local haematoma or bruise and some participants may report pain or discomfort.

All performed functional measurements of EF are non-invasive and risk are therefore minimal. During the evaluation of the macro vascular blood circulation by FMD, participants will experience a slight tingling in the arm for a small period of time, because during a FMD the blood flow is temporarily obstructed. During the evaluation of the micro vascular circulation, participants might (not in all cases) feel a small prickling during the first minute on their skin, which is caused by the small electric current. The time investment requested from the participants is shown in table 5 and is set at 56 hours for each individual.

**Table 5:** *Time investment per participant*

|  | Time at University | Questionnaires | Weight /height | Blood collection during screening | total |
| --- | --- | --- | --- | --- | --- |
| Information meeting | 1.5 hours |  |  |  | 1.5 hrs. |
| screening |  | 15 min | 5 min | 10 min | 0.5 hrs. |
| Intervention HF | 9 hours |  |  |  | 9 hrs. |
| Intervention HF | 9 hours |  |  |  | 9 hrs. |
| Intervention HF | 9 hours |  |  |  | 9 hrs. |
| Intervention ‘control’ | 9 hours |  |  |  | 9 hrs. |
| Intervention ‘control’ | 9 hours |  |  |  | 9 hrs. |
| Intervention ‘control’ | 9 hours |  |  |  | 9 hrs. |
|  |  |  |  | Total time whole study | 56 hrs. |

**9.5 Compensation for injury**

Wageningen University has a liability insurance which is in accordance with article 7, subsection 6 of the WMO.

Wageningen University (also) has an insurance which is in accordance with the legal requirements in the Netherlands (Article 7 WMO and the Measure regarding Compulsory Insurance for Clinical Research in Humans of 23rd June 2003). This insurance provides cover for damage to research subjects through injury or death caused by the study.

1. € 450.000,-- (i.e. four hundred and fifty thousand Euro) for death or injury for each participant who participates in the Research;

2. € 3.500.000,-- (i.e. three million five hundred thousand Euro) for death or injury for all participants who participate in the Research;

3. € 5.000.000,-- (i.e. five million Euro) for the total damage incurred by the organization for all damage disclosed by scientific research for the Sponsor as ‘verrichter’ in the meaning of said Act in each year of insurance coverage.

The insurance applies to the damage that becomes apparent during the study or within 4 years after the end of the study.

Wageningen university has this insurance at:

Gerling-Konzern

Allgemeine Versicherungs-AG

Directie voor Nederland

Herengracht 520

1017 CC Amsterdam

**10. ADMINISTRATIVE ASPECTS AND PUBLICATION**

**10.1 Handling and storage of data and documents**

Before the start of the study, participants will be assigned a random number that will not change during the study. This number is linked with the name, address, date of birth, and telephone number of the participant in a password-protected file. Only members of the project team can access this file. For all other purposes, the random number will be used for participant identification. The informed consents will be stored separately from all other information. After the end of the study the link between the identity of the participants and the assigned numbers will be destroyed. Only means and other statistical expressions based on individual data will be published. Human material, which is sampled during the study and stored anonymously, will be destroyed 12 years after the end of the study.

**10.2 Amendments**

Amendments are changes made to the research after a favorable opinion by the accredited METC has been given. All amendments will be notified to the METC that gave a favorable opinion.

**10.3 End of study report**

The investigator will notify the accredited METC of the end of the study within a period of 8 weeks. The end of the study is defined as the last day of the intervention. In case the study is ended prematurely, the investigator will notify the accredited METC, including the reasons for the premature termination.

**10.4 Public disclosure and publication policy**

Publication policy is in agreement with the CCMO publication statement. Nor the sponsors, nor the principal investigator has a right of veto regarding the way of publishing the results. Partners/ sponsors have the right to take cognizance of the results in order to patent some of the outcomes (if applicable).

**11. REFERENCES**

1. Kuvin, J.T. et al. Peripheral vascular endothelial function testing as a noninvasive indicator of coronary artery disease. *J Am Coll Cardiol* **38**, 1843-1849 (2001).

2. Cai, H. & Harrison, D.G. Endothelial dysfunction in cardiovascular diseases: the role of oxidant stress. *Circ Res* **87**, 840-844 (2000).

3. Khazaei, M., Moien-Afshari, F. & Laher, I. Vascular endothelial function in health and diseases. *Pathophysiology* **15**, 49-67 (2008).

4. van Oostrom, A.J. et al. Postprandial recruitment of neutrophils may contribute to endothelial dysfunction. *J Lipid Res* **44**, 576-583 (2003).

5. Van Oostrom, A.J., Sijmonsma, T.P., Rabelink, T.J., Van Asbeck, B.S. & Cabezas, M.C. Postprandial leukocyte increase in healthy subjects. *Metabolism* **52**, 199-202 (2003).

6. Keogh, J.B., Grieger, J.A., Noakes, M. & Clifton, P.M. Flow-mediated dilatation is impaired by a high-saturated fat diet but not by a high-carbohydrate diet. *Arterioscler Thromb Vasc Biol* **25**, 1274-1279 (2005).

7. Ceriello, A. et al. Evidence for an independent and cumulative effect of postprandial hypertriglyceridemia and hyperglycemia on endothelial dysfunction and oxidative stress generation: effects of short- and long-term simvastatin treatment. *Circulation* **106**, 1211-1218 (2002).

8. Vogel, R.A., Corretti, M.C. & Plotnick, G.D. Effect of a single high-fat meal on endothelial function in healthy subjects. *Am J Cardiol* **79**, 350-354 (1997).

9. Westphal, S. et al. Endothelial dysfunction induced by postprandial lipemia is neutralized by addition of proteins to the fatty meal. *Atherosclerosis* **185**, 313-319 (2006).

10. van Oostrom, A.J. et al. Activation of leukocytes by postprandial lipemia in healthy volunteers. *Atherosclerosis* **177**, 175-182 (2004).

11. De Roos, N.M., Bots, M.L., Schouten, E.G. & Katan, M.B. Within-subject variability of flow-mediated vasodilation of the brachial artery in healthy men and women: implications for experimental studies. *Ultrasound Med Biol* **29**, 401-406 (2003).

12. Schalkwijk, C.G. & Stehouwer, C.D. Vascular complications in diabetes mellitus: the role of endothelial dysfunction. *Clin Sci (Lond)* **109**, 143-159 (2005).

13. Abahji, T.N. et al. Acute hyperhomocysteinemia induces microvascular and macrovascular endothelial dysfunction. *Arch Med Res* **38**, 411-416 (2007).

14. Murohara, T., Buerke, M. & Lefer, A.M. Polymorphonuclear leukocyte-induced vasocontraction and endothelial dysfunction. Role of selectins. *Arterioscler Thromb* **14**, 1509-1519 (1994).

15. Giannattasio, C. et al. Fluctuations of radial artery distensibility throughout the menstrual cycle. *Arterioscler Thromb Vasc Biol* **19**, 1925-1929 (1999).

16. Hashimoto, M. et al. Modulation of endothelium-dependent flow-mediated dilatation of the brachial artery by sex and menstrual cycle. *Circulation* **92**, 3431-3435 (1995).

17. Williams, M.R. et al. Variations in endothelial function and arterial compliance during the menstrual cycle. *J Clin Endocrinol Metab* **86**, 5389-5395 (2001).

18. Bots, M.L., Westerink, J., Rabelink, T.J. & de Koning, E.J. Assessment of flow-mediated vasodilatation (FMD) of the brachial artery: effects of technical aspects of the FMD measurement on the FMD response. *Eur Heart J* **26**, 363-368 (2005).

19. Stehouwer, C.D., Henry, R.M. & Ferreira, I. Arterial stiffness in diabetes and the metabolic syndrome: a pathway to cardiovascular disease. *Diabetologia* **51**, 527-539 (2008).
